# A flexible systems analysis pipeline for elucidating spatial relationships in the tumor microenvironment linked with cellular phenotypes and patient-level features

**DOI:** 10.3389/fimmu.2025.1642527

**Published:** 2025-09-29

**Authors:** Gabriel F. Hanson, Kate A. Goundry, Remziye E. Wessel, Michael G. Brown, Timothy N. J. Bullock, Sepideh Dolatshahi

**Affiliations:** ^1^ Department of Biomedical Engineering, University of Virginia, Charlottesville, VA, United States; ^2^ Beirne B. Carter Center for Immunology Research, University of Virginia School of Medicine, Charlottesville, VA, United States; ^3^ Department of Medicine, Nephrology, University of Virginia School of Medicine, Charlottesville, VA, United States; ^4^ Center for Immunity, Inflammation and Regenerative Medicine, University of Virginia School of Medicine, Charlottesville, VA, United States; ^5^ Department of Microbiology, Immunology and Cancer Biology, University of Virginia School of Medicine, Charlottesville, VA, United States; ^6^ Department of Pathology, University of Virginia School of Medicine, Charlottesville, VA, United States

**Keywords:** spatial biology, spatial proteomics, supervised machine learning, T cell, NK cell, immune interactions, tumor-immune cell interactions, systems immunology

## Abstract

**Introduction:**

Quantitative investigation of how the spatial organization of cells within the tumor microenvironment associates with disease progression, patient outcomes, and that cell’s phenotypic state remains a key challenge in cancer biology. High-dimensional multiplexed imaging offers an opportunity to explore these relationships at single-cell resolution.

**Methods:**

We developed a computational pipeline to quantify and analyze the neighborhood profiles of individual cells in multiplexed immunofluorescence images. The pipeline characterizes spatial co-localization patterns within the tumor microenvironment and applies interpretable supervised machine learning models, specifically orthogonal partial least squares analysis (OPLS), to identify spatial relationships predictive of cell states and clinical phenotypes.

**Results:**

We applied this framework to a previously published non-small cell lung cancer (NSCLC) cohort across four applications. At the cellular level, we identified neighborhood features associated with lymphocyte activation states. At the tumor-immune interface, we demonstrated that the immune cell composition surrounding major histocompatibility complex class I-expressing (MHC I^+^) tumor cells could distinguish adenocarcinoma from squamous cell carcinoma. At the patient level, spatial features predicted tumor grade.

**Discussion:**

By integrating cell-segmented imaging data with interpretable modeling, our pipeline reveals key spatial determinants of tumor biology. These findings generate testable mechanistic hypotheses about intercellular interactions and support the development of spatially informed prognostic and therapeutic strategies.

## Graphical Abstract

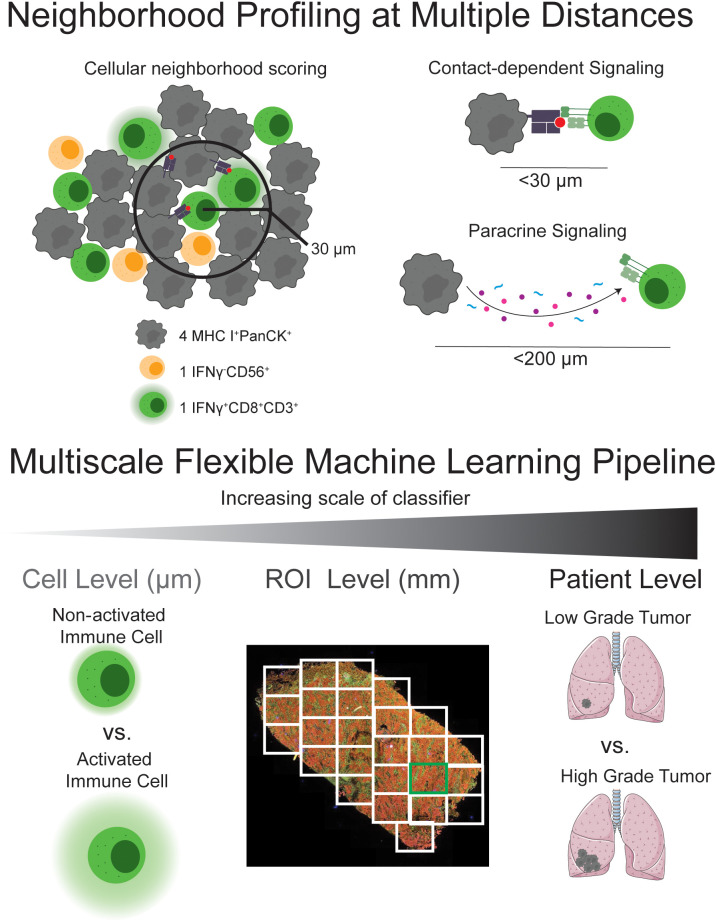

## Introduction

The tumor microenvironment (TME) encompasses the cellular landscape surrounding a tumor, including malignant cells, immune cells, stromal cells, extracellular matrix, vasculature and lymphatics, and signaling molecules whose interplay shapes tumor progression, therapeutic response, and patient outcomes. Recent advancements in imaging and transcriptomic sequencing have revealed the heterogeneity of the TME, both in composition and spatial arrangement (1). However, further methodological development is needed to effectively integrate and interpret these complex, high dimensional spatial data. This gap limits our ability to extract mechanistic insights and identify predictive biomarkers from emerging spatial multi-omics data, motivating the framework we introduce here.

Direct tumor-immune interactions depend on the spatial proximity of immune cells to the tumor cells and to each other. Immune cells play diverse and sometimes opposing roles in the TME, depending on their type, activation state, and context. Certain immune cells, such as cytotoxic T cells and natural killer (NK) cells can directly attack and kill malignant cells. Others, including regulatory T cells and some macrophage subsets, may instead support tumor growth by suppressing anti-tumor immunity or promoting tissue remodeling. In addition to interacting with tumor cells, immune cells also engage with one another to coordinate immune responses. These immune-immune interactions can activate effector functions, shape pathways, and influence the local cytokine milieu (2–4). Depending on the nature of the signals exchanged, these interactions can either enhance anti-tumor activity or contribute to immunosuppressive environments. Interactions between cells of the TME occur by way of both cell surface receptor interactions and secreted signaling molecules, necessitating the proximity of interacting cells at distances relevant for juxtracrine and paracrine signaling, respectively (5). Observing cell neighbors within a radius corresponding to the diameter of a typical cell (between 5-20 µm) may reveal the potential for cell surface receptor interactions and expanding analysis to 200-250 µm reveals cells within paracrine signaling limits (6).

Advancements in multiplex imaging techniques such as multiplexed ion beam imaging (MIBI) (7, 8), co-detection by indexing (CODEX) (9, 10), imaging mass cytometry (IMC) (11, 12), and multiplexed immunofluorescence (mIF) imaging or multiplexed immunohistochemistry (mIHC) (13–15) enable simultaneous protein phenotyping of cells within tumor regions while preserving spatial architecture. In this study, we explored spatial co-localizations using mIF, though this systems framework may be applied to data gathered from other spatial proteomic and transcriptomic techniques.

Recent studies highlight the efficacy of spatial molecular data in predicting response to immunotherapy, especially colocalization analysis (16, 17). A wide range of analytical tools have emerged in response to these advances in spatial profiling technologies, including ecological packages like Spatstat (18), vegan (19), and ecoCopula (20), as well as domain-specific frameworks such as Spatial TIME (21), MonkeyBread (22), CELESTA (23), Crescendo (24), and SPIAT (25). These tools have collectively expanded the analytical landscape by introducing novel conceptual frameworks and demonstrating proof-of concept strategies for quantifying intercellular spatial relationships within the TME cells. This growing toolkit has enabled a more precise characterization of the spatial organization of the TME – referred to as the *colocatome* (26)- and has deepened our understanding of intratumoral cellular interactions (27). More recently, frameworks such as CellLENS (28), MONTAGE (29), and MicroCart (30) have further advanced the field by integrating multiple spatial domains including expression, neighborhood context, and tissue localization to uncover clinically relevant immune populations, functional cellular communities, and host–microbiome spatial dynamics, respectively.

Spatial analysis has proven effective for elucidating anti-tumor immune mechanisms, predicting treatment responses, and evaluating prognostic outcomes. For instance, Sudmeier et al. synthesized spatial localization data about CD8^+^ T cells with phenotypic and transcriptomic data in glioblastoma to investigate signaling pathways at the tumor-immune interface (31). In metastatic melanoma, the spatial proximity of PD-1^+^ and PD-L1^+^ was associated with improved response to immunotherapy (32). In non-small cell lung cancer (NSCLC), anti-PD1 treatment was shown to induce localized “multicellular immunity hubs” with favorable clinical outcomes (17), and spatial profiling was recently used to reveal that TIM-3 expression is enriched in precancerous lesions and lost during LUAD progression, suggesting a role for TIM-3 in early immune evasion and as a target for interception strategies (33).

Despite these successes, the high dimensionality of modern spatial data presents new analytical challenges. As spatial profiling technologies improve and increasingly complex data is produced, multivariate methods have become essential tools for interpreting the spatial heterogeneity in the TME. Many of these methods leverage machine learning, to deconvolute complex data and to create clinically relevant predictions (34). While deep learning approaches have grown in popularity for these tasks, their black-box nature often limits biological interpretability. In contrast, supervised statistical learning approaches (e.g., partial least squares discriminant analysis, PLS) offer transparent, interpretable models that remain valuable for hypothesis generation and mechanistic insight. However, machine learning applications in spatial multi-omics remain underdeveloped, in part because analytical frameworks have not kept pace with rapid advances in spatial technologies. As a result, it is unclear how to best structure spatial data for analysis, which spatial features are most biologically meaningful or predictive, and what types of questions can be rigorously addressed using these tools.

To address this gap, we designed a systems analysis framework to identify and characterize neighborhood colocalization profiles across multiple scales, from single cells to patient-level features. Our approach responds to the need for interpretable, multivariate models that can integrate spatial complexity into testable biological and clinical hypotheses. This framework builds on existing neighborhood quantification methods by incorporating rigorous statistical pipelines and emphasizing biological interpretability. By leveraging single cell-resolved neighborhood profiles and multivariate analysis, we enhance the ability to accurately classify cell and tumor states from the micrometer scale up to patient characteristics. Here, we introduce this novel analysis framework, offer recommendations for data handling and quality control, and demonstrate its applicability with examples from a previously published mIF dataset of NSCLC biopsies (35).

## Methods

### Tissue specimens and data summary

As described before (35), this study included a cohort of 36 NSCLC patients with resected tissue, including 4 who received immune checkpoint immunotherapy (ICI) at the University of Virginia between 2014 and 2018 (**Table 1**). Multispectral fluorescence imaging using the PerkinElmer Vectra 3.0 imaging system was performed on these tissue samples. Quality control measures were first performed including inspection of fluorescence intensity distributions and verification of marker positivity thresholds, exclusion of ROIs with low cell counts, and confirmation that all ROIs and cells are uniquely identified (see below for more details). Additionally, four patients were excluded from downstream analysis due to either missing metadata or aberrant CD56 expression on malignant cells, which confounded our use of CD56 as a marker for NK cells. This resulted in a final cohort of 32 NSCLC patients for analysis and characterization.

**Table 1:** NSCLC cohort characteristics *mean ± SD (minimum-maximum).

NSCLC cohort (n=32)	Adenocarcinoma (LUAD) (n=26)	Squamous cell carcinoma (LUSC) (n=6)
Dead/Alive (% alive)	5/21 (80.7%)	2/4 (66.7%)
Recurrence	9	2
Female/Male	17/9	3/3
Age*	67.7 ± 9.9	61.8 ± 9.1
Cancer Grade (G1/G1-G2/G2/G2-G3/G3/G4)	5/1/9/1/10/0	0/0/1/4/0/1
Largest Tumor Dimension* in cm (Range)	3.41 (<2 – 6.3)	3.43 (1.8-5.5)
Treated with Immunotherapy	4	0

### mIF imaging and geospatial analysis

NSCLC patient tissues were analyzed as described in (35). Briefly, they were stained with antibodies against pan-cytokeratin (PanCK), the HC10 monoclonal antibody which is a pan- major histocompatibility complex class I (MHC I) antibody, staining the MHC class I (MHC I) alleles HLA-A, -B, and -C, CD3, CD8, CD56, interferon gamma (IFNγ), and DAPI. HALO (Quantitative Image Analysis for Pathology by Indica Labs) was used to segment individual cells and assign stain positivity. Combinatorial marker expression was used to classify cellular phenotypes (**Table 2**), and a classifier was trained to identify tumor and stroma regions based on PanCK positivity. Each cell was then assigned a specific phenotype and regional annotation for downstream analysis.

**Table 2:** Cell classification markers.

Cell phenotype	Phenotypic markers
Tumor Cell	PanCK^+^ MHC I^+^
Tumor Cell with MHC I Loss	PanCK^+^ MHC I^-^
Cytotoxic T Cell	PanCK^-^ CD3^+^ CD8^+^
CD8^-^ T Cell	PanCK^-^ CD3^+^ CD8^-^
Natural Killer (NK) Cell	PanCK^-^ CD3^-^ CD56^+^
Immune Cell Activation Marker	IFNγ

### Cellular neighborhood scoring

#### Cell neighborhood vector quantification

An in-house custom algorithm, as described and employed by us previously (35) was used to construct neighborhood profiles. Briefly, intercellular spatial colocalizations in NSCLC tumors were determined in Python using 2-dimensional coordinates of individual segmented cells obtained from HALO. The Euclidian distance between each cell and every other cell on the slide was computed; r-neighbors were defined as cells with a center-to-center Euclidian distance of less than the user-specified radius r from the center cell. The r-neighbors of each phenotype were enumerated to yield the neighborhood profile for every individual cell. We refer to the individual count of neighboring cells of a specific phenotype (*e.g.* CD8 neighbors around a tumor cell) as the neighborhood score. The complete set of scores across all phenotypes forms the neighborhood vector for that cell, representing its spatial context in a structured interpretable format. The mathematical formulation is described below:

Let: $P=\left\{p_{1},p_{2},\ldots ,p_{m}\right\}$ denote a set of user-defined phenotypic labels.
Let: $C=\left\{c_{1},c_{2},\ldots ,c_{n}\right\}$ be the set of all cells, where each cell c∈ C has:
Spatial coordinates $X_{c}\in {\mathbb{R}}^{2}$ ,
A phenotype label l(c)∈P .
  Let $R=\left\{r_{1},r_{2},\ldots ,r_{K}\right\}$ be a set of radii of interest.

For each queried phenotype $p_{i}\in P$ , Equation 1 defines the subset of queried cells:


(1) $$C_{p_{i}}=\{c\;\in \;C:\ell (c)=p_{i}\}$$

In Equation 2 we define the neighborhood score, a transformation $T_{P}^{r}\left(c\right):\;C\to \;{\mathbb{N}}^{m}$ that maps a center cell c ∈C to a count of neighboring cells of each phenotype *p_i_* ∈P within a fixed radius r, which makes an m-dimensional vector, ℕ^*m*^ where ℕ is defined here as the set of non-negative integers:


(2) $$T_{p_{i}\;}^{r}\left(c\right)=\;\sum_{\begin{array}{l} c^{\prime }\in \;C\\ c^{\prime }\neq c \end{array}}1(∥X_{c^{\prime }}-X_{c}∥\leq r)*1\left(\ell \left(c^{\prime }\right)=p_{i}\right)$$

Where:

*c'* is a neighboring cell

*X_c_* and *X_c'_* are the spatial coordinates of the center and neighbor cells respectively

1 (…) is an indicator function that maps the elements of a subset to one, and all other elements to zero.

Thus, we define the neighborhood vector for each cell in Equation 3:


(3) $$T^{r}=\;\left[T_{p_{1}}^{r}(c),T_{p_{2}}^{r}(c)\;,\;\ldots ,T_{p_{m}}^{r}(c)\;\right]$$

Throughout this manuscript, this neighborhood vector was calculated for $ℛ=\left\{r_{1},r_{2}\right\}=\left\{30\;\left[um\right],\;200\;\left[um\right]\right\}$ for cell contact and paracrine signaling, respectively. These radii can be adjusted to accommodate cell size and based on specific known intercellular signaling ranges. These neighborhood vectors were inputted to downstream supervised multivariate analysis.

#### Intercellular K-function analyses

Ripley’s K function is a spatial statistics tool used to evaluate whether the spatial distribution of points – here, cells and their phenotypic subsets – deviates from complete spatial randomness. It assesses whether cells tend to cluster, repel, or distribute independently, which can provide insight into underlying mechanisms of coordination. The cross K-function extends this analysis to two distinct phenotypic subsets, quantifying their degree of spatial colocalization or segregation in a pairwise manner. Here spatial colocalization patterns in NSCLC tumors were further analyzed using the *Spatstat* package (18) in R version 4.2.1 to yield the cross K-function traces to describe the colocalization patterns of pairs of cell types of interest at a continuous range of radii up to 200μm and compare it with a completely random placement of cells (homogeneous Poisson) process as the null model. This continuous range analysis complements the previously described neighborhood vector calculations. The codes for this analysis are available at Dolatshahi-lab GitHub (https://github.com/Dolatshahi-Lab/NSCLC_SpatialMethods) and data are available on LibraData: (https://doi.org/10.18130/V3/VQFO1J).

### Quality control and pre-processing

Distances were first converted from pixels to micrometers using HALO informed conversion ratios. Staining positivity thresholds were verified by plotting histograms of cell staining intensities along with cutoffs for positivity, consistent across tissue samples. Phenotypic labels were defined by biologically informed protein marker combinations (**Table 2**). After labeling and phenotypic enumeration, cell densities within 3 mm^2^ regions of interest (ROIs) were summarized by percent composition. Quality control was performed by removing ROIs where more than 90% of cells were unlabeled as well as ROIs with less than 500 cells. Additionally, three patients had tumors that stained positive for PanCK as well as CD56, our marker for NK cells. These patients were also removed. After this quality control and filtering we were left with 414 ROIs composed of 5307540 cells belonging to 33 patients. One of these patients lacked histological metadata and was included in cell level analysis but removed in patient level analysis. For grade analysis, patients were further filtered to just those with a histological classification of Adenocarcinoma.

### Normalization

After computing neighborhood scores for each cell of interest, the resulting neighborhood scores were log-transformed using log(1 + p) scaling to reduce the impact of extreme values. These neighborhood scores were then z-scored across each phenotypic neighborhood label. The normalized matrix of neighborhood scores was subsequently used as input for building partial least squares discriminant analysis (PLSDA) models.

In cases where cells are being compared between different tumors or ROIs, which often have different overall immune cell densities, cell relationships may be inflated by the density of each cell type which may not represent differences in interactions and “intentional” cellular colocalization. Normalization might be necessary if enrichment of interactions is being assessed. As such, for tumor- or ROI-level analysis such as those in Application 4, an additional level of normalization was performed. These neighborhood vectors were first calculated as described before (see cellular neighborhood scoring) and then were further normalized by dividing by the total number of center cells ( $N_{c}$ ) of a certain phenotypic label (*p_i_*) in each ROI as well as by the square root of the number of neighbors ( $N_{n}$ ) of a certain phenotypic label in that ROI (*p_j_*) (Equation 4). The division by *N_c_* calculates the average per ROI or patient. The division by $\sqrt{N_{n}}$ accounts for the cell type-specific density bias. This was repeated for each phenotypic pair, resulting in neighborhood scores for each pairwise combination of center and neighbor cells. The resulting matrix of normalized neighborhood scores by ROI was then z-scored and used as the input to the PLSDA models.


(4) $$\begin{array}{c} Normalized\;Neighborhood\;Score\\ \;=\;\frac{1}{N_{c}}\frac{1}{\sqrt{N_{n}}}\sum_{c\in p_{i}}T_{p_{j}\;}^{r}\left(c\right) \end{array}$$

### Multivariate discriminate analysis and sampling techniques

Partial Least Squares (PLS) is a powerful supervised linear machine learning approach that has been widely used to identify the top contributors to a continuous outcome of choice or group differences, called PLS regression (PLS-R) and PLS discriminant analysis (PLS-DA), respectively. Orthogonalized PLS (OPLS) builds upon this framework by orthogonalizing the model such that variation in Y is captured on a single latent variable (LV) and other latent variables described variance orthogonal (not contributing) to Y for ease of interpretation. Here, OPLS models were generated in Python using a combination of the *pyopls* package for orthogonalization and *PLSRegression* function in the scikit-learn package (36). For OPLS-DA models, the 5-fold cross validation (CV) accuracy was reported, and significance was calculated by comparing the CV accuracy of the constructed model against the 5-fold CV accuracy of 1000 OPLS-DA models based on data with randomly shuffled labels to compute an empirical permutation testing p-value. For OPLS-R models, significance was calculated empirically by comparing the constructed model’s mean squared error (MSE) and CV Q^2^ to 1000 OPLS-R models based on data with randomly permuted continuous labels (37, 38). Variable importance in projection (VIP) scores were used to rank the predictors in their contribution to the response variable. A VIP score quantifies the contribution of each variable to the PLS-DA or PLS-R model, summarizing how influential each predictor is in explaining the variance of the response variable. Variables with higher VIP scores are considered more relevant for group separation (PLS-DA) or response prediction (PLSR), and VIP scores are commonly used to guide feature selection. In practice, variables with a VIP score greater than 1 are typically regarded as contributing more than average to the response variable (39).

For patient level analysis where a large number of predictor variables (here after called features) are involved, we applied the least absolute shrinkage and selection operator (LASSO) (40) for feature selection, followed by classification using only the features selected by LASSO. We used the *LassoCV* function from sklearn with 10-fold cross validation to determine the optimal regularization parameter (alpha). A correlation network was then constructed to identify and visualize additional variables that were highly correlated (|r| > 0.75) with those selected by LASSO.

Model performance was additionally evaluated using F1 scores and area under the receiver operating characteristic curve (AUROC) values. The F1 scores provide a comprehensive metric encompassing both precision and recall, thereby accounting for class imbalance. AUROC quantifies the model’s ability to distinguish between classes across all classification thresholds.

### Downsampling and bootstrapping for balanced and robust modeling

When discriminant analysis is performed on imbalanced datasets, where the distribution of the target variable is skewed toward one class (*e.g.* Application 2), the resulting model may be biased toward the majority class. This is a common issue in cell-level analysis. To address this, we employed stratified downsampling combined with bootstrapping to ensure balanced representation of the classes and quantify variability in model performance.

Specifically, for cell level analysis, the overrepresented center cell phenotype was downsampled prior to modeling, matching the minority group in sample size (defaulting to 50% of the smaller group). This resampling was repeated across multiple bootstrap iterations (approximately 10 iterations per sample), generating a distribution of model accuracies and VIP scores. This bootstrapped distribution enabled us to calculate variability metrics (*e.g.* standard deviation, confidence intervals), providing a more reliable measure of model robustness. Across applications, permutation testing p-values, precision, and F1 scores improved, indicating a better overall fit to the data.

For regression tasks, we similarly applied bootstrapping; a fixed number of samples were randomly drawn with replacement in each iteration to fit the OPLS models. Model performance was assessed using 5-fold cross validation to compute mean squared error (MSE) and Q^2^ scores.

To determine the optimal bootstrapping parameters, we conducted sensitivity analysis varying sample size and number of iterations while holding the dataset coverage constant (10x). This analysis revealed that model stability, defined as convergence of performance metrics (*e.g.* cross-validation accuracy, MSE) and low variance in feature importance rankings (VIP scores) depends on dataset size and heterogeneity. By this analysis we selected sample sizes of 3705, and a bootstrap iteration number of 2001 for the regression model and 50% of the smaller group size for classification models to ensure reliability and reproducible estimates.

We also applied down-sampling in region-level analyses and spatial statistics to address non-independence among observations. For patient-level comparisons, we matched the number of ROIs sampled per patient to 50% of the minimum number across the cohort, repeatedly subsampling to incorporate more data while maintaining class balance. For cell-dense phenotypes, center cells were down-sampled to mitigate the statistical challenges that arise when neighboring cells share microenvironmental features and thus cannot be treated as independent. The number of sampled cells per ROI was thus matched to the number of approximately non-overlapping neighborhoods that could fit within the 3mm by 3mm ROI. This corresponds to roughly 500–700 cells for a 30μm radius and 15–20 center cells per ROI for a 200μm to minimize spatial overlap and ensure improved independence among observations.

For groups comparisons, Mann-Whitney U tests were employed as a non-parametric method, offering a robust alternative to parametric tests when data do not meet normality assumptions. However, recognizing that traditional statistical methods may still be influenced by spatial dependencies, permutation testing was also be implemented as an alternative approach. In permutation testing, the true neighborhood score is compared to a null distribution of neighborhood scores generated from randomized cell labels. This method provides a flexible, data-driven framework that accounts for underlying spatial structures and reduces the risk of false positive findings.

### Recommendations for spatial colocalization analysis

We compiled a table of best practices based on our experiences with spatial single cell-resolved multiplexed data such as multiplex immunofluorescence imaging (**Table 3**), which can be applied to other spatial proteomics and transcriptomics data. For each step, the table outlines specific recommendations, provides the rationale behind them, and lists implementable tools and packages. We hope this resource will serve as a resource for effectively managing and analyzing similar data and building upon.

## Results

**Figure 1:**
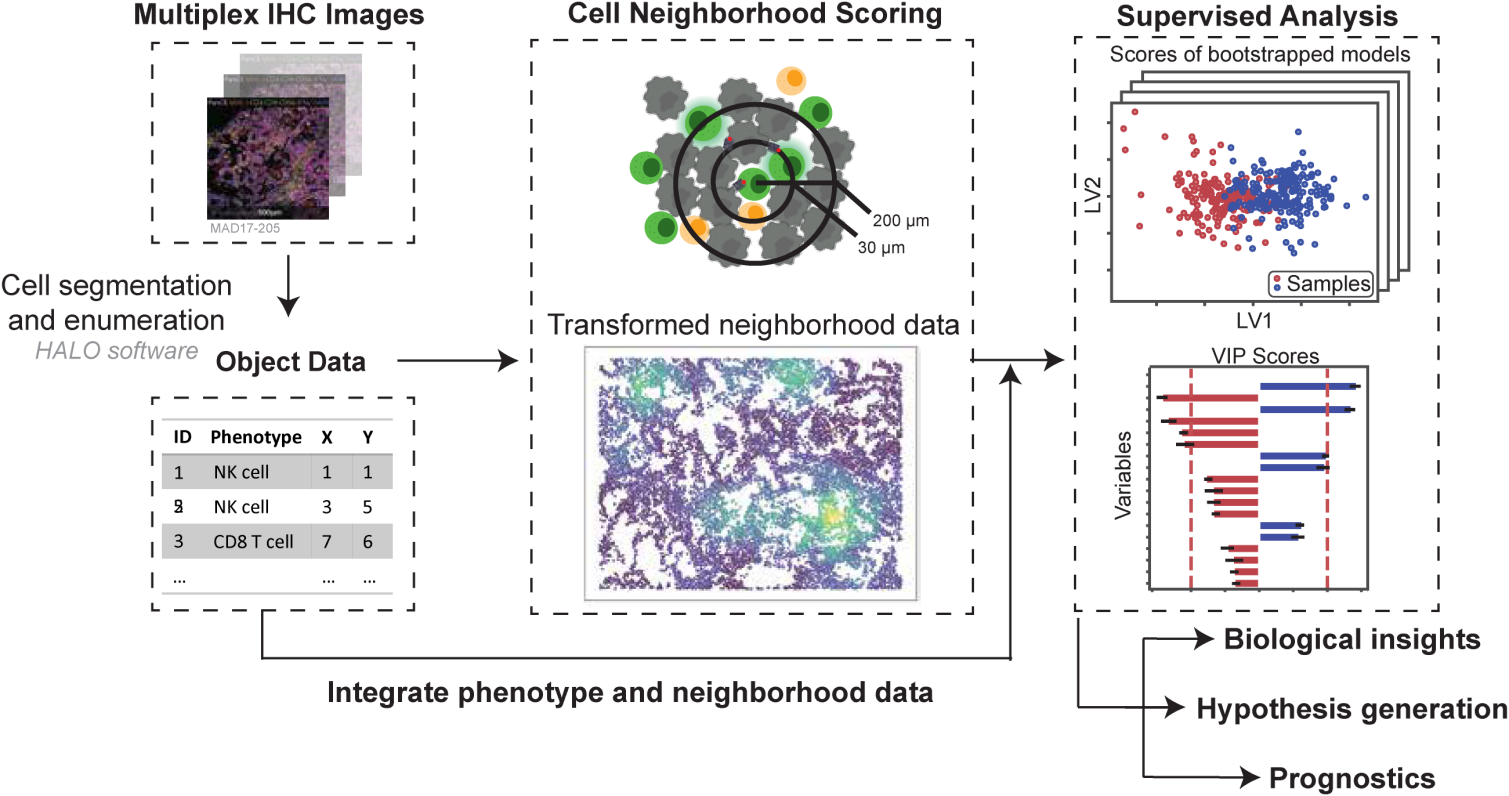
Pipeline overview - Cell ecological feature extraction from mIF data combined with supervised multivariate analysis predicts cellular- and cohort-level phenotypes. Cell segmentation is performed on mIF images and staining positivity is assigned, digitizing the images. Neighborhood profiles are calculated for each cell by quantifying the number of cells of each phenotype at distances relevant for juxtacrine and paracrine signaling. Combinations of immunofluorescent stains are used to assign phenotypes to cells across the images. Bootstrapped Partial least squares analysis identifies spatial relationships between cell types that predict cellular characteristics (e.g., activation) and cohort-level outcomes (e.g., survival, histological type) based on neighborhood profiles. These relationships are validated using univariate approaches. This method is based on the hypothesis that multiplexed spatial proteomic data contain granular, pairwise spatial relationships that, when systematically analyzed, can reveal meaningful patterns of cellular colocalization and function. We analyze spatial data at single-cell resolution by quantifying, for each “center” cell, the number of “neighbor” cells of each phenotype within a defined radius. This process yields a local neighborhood profile for every cell in the tissue, analogous to applying a moving average filter across the spatial domain. Radii are selected to reflect biologically relevant distances for contact-dependent and paracrine signaling.

Our pipeline is a two-step process that takes single-cell spatial data as input, first quantifying the local cellular neighborhoods around each cell, and then applying multivariate statistical analysis to uncover spatial patterns and associations (**Figure 1**). Using a previously published mIF dataset of NSCLC tumors (35) multivariate statistical models were built to link quantified cell-cell colocalizations to cellular and clinical metrics across scales spanning cell state (Application 1 and 2) to tissue- and patient-level characteristics such as histological subtype or tumor grade (Applications 3 and 4). We provide strategies for data pe-processing and normalization, choice of quantitative spatial metrics and radii around each cell, as well as suggestions for rigorous data analysis practices and goodness of fit criteria (**Table 3** – Best practices).

**Table 3:** Pipeline summary and recommendations.

Stage	Recommendations	Rationale	Tools
A. Data Collection	Use single cell-segmented data with spatial coordinates and multiplexed measurements (proteins or transcripts).	Enables spatial mapping of diverse cell types and quantification of interactions.	Segmentation algorithms: Cellpose (41), CellProfiler (42), HALO by Indica labs
B. Preprocessing	Apply log normalization to neighborhood scores.	Improves distributional properties and downstream model assumptions (*e.g.*, Gaussianness, homoscedasticity).	Q-Q plots, KS tests
C. Radius selection	Choose biologically relevant radii (*e.g.*, r = 30 um for direct contact; 200 um for cytokine-mediated paracrine signaling) based on the sizes of cell types and suspected modes of communications. Perform sensitivity analysis to the choice of r.	Captures communication at relevant scales and ensures robustness to the choice of radius, while enabling biological interpretability.	Euclidean distance for spatial proximity, for neighborhood scoring.
D. Cross K-function	Use cross K-function (or G-function or others from the theory of point processes) across a range of radii in addition to discrete metrics.	Summarizes interactions at multiple scales; enables comparison to null, e.g. Poisson, models.	Ripley’s K-function via Spatstat and SPIAT (18, 25, 43)
E. Phenotype selection	Use biologically relevant cell types; separate tumor/stroma (or additional tissue context) when possible.	Prevents misclassification; improves interpretability and biological relevance.	Morphological classifiers
F. Density Normalization	For tissue or patient-level characteristics, normalize neighbor counts by overall density.	Controls for sample-to-sample variation in cell abundance and density.	Equation 4. (Normalized neighborhood score)
G. Down sampling/Bootstrapping	Balance group sizes by subsampling with replacement (bootstrap) and calculate accuracy and VIP scores across iterations. Use sensitivity analysis to guide sample size and iteration count based on dataset size and heterogeneity. Sampling half the smaller class (classification) or a fixed fraction of the dataset (regression), with ~10× total sample coverage, yields stable performance- defined as convergence of accuracy estimates and low variance in feature rankings (VIP scores) across iterations.	Prevents class imbalance effects; supports robust model evaluation, allows model and feature importance errors to be estimated.	Python scikit-learn (36), imbalanced-learn
H. Feature Selection	Use LASSO or Elastic Net to select inputs; recover correlated features via correlation networks.	Reduces overfitting in high-dimensional settings, e.g., when comparing pairwise relationships across multiple cell types for tissue-level comparisons; recovers correlated features excluded by regularization.	Python scikit-learn (LASSO/Ridge/ Elastic Net), Cytoscape (44) for network visualization
I. Supervised Modeling	Use interpretable linear model (*e.g.*, OPLS-DA) or nonlinear classifiers as appropriate.	Balances performance with interpretability; handles multicollinearity; ranks features based on their contribution to effect.	Python scikit-learn (PLSRegression),R (MixOmics package) (45)
J. Validation	Use cross-validation (*e.g.*, 5-fold); validate on external cohort if possible.	Improves generalizability and guards against overfitting.	Python scikit-learn (CV module)
K. Univariate Testing	Use univariate statistics on top features identified by the multivariate models with caution to spatial dependence between single cell samplings of neighborhood scores.	Complements multivariate models but requires careful statistical handling.	See Wilson et al. (46) for spatial testing considerations

**Figure 2:**
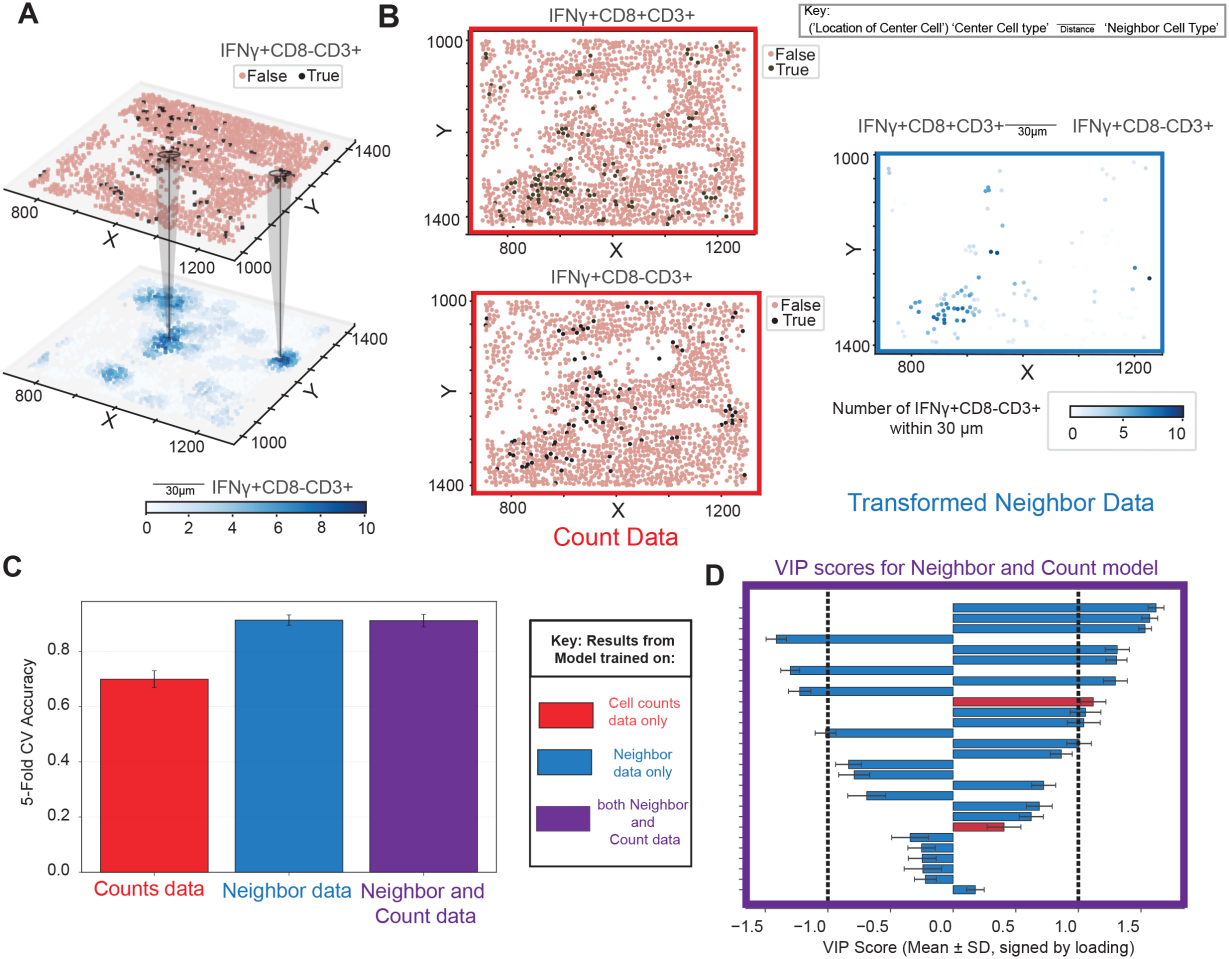
Neighborhood colocalization transformation creates single cell data with predictive power. **(A)** Non-transformed count data highlighted in red. **(B)** Spatial point data is transformed by counting neighbors of a given cellular phenotype within a given radius. This results in a transformation quantifying the pairwise relationships between phenotypes within a radius on a cell-by-cell basis. Transformed neighbor data highlighted in blue. **(C)** Cross-validation accuracy of predictive models trained to identify which patients had high MHC class I expression on tumor cells based on 3 sets of different input features: non-transformed cell counts data (red), neighborhood-transformed data (blue), or a combination of both (purple). Bars represent one standard deviation around the mean accuracy across bootstraps. **(D)** Variable importance in projection (VIP) scores from the model that contains a mixture of counts features and neighbor relationships, underscoring the added value of including cellular colocalization, which appear on top of the list. Throughout the manuscript VIP scores are artificially assigned the sign of the loading of that feature on LV1 to visually highlight the group they are higher in. Error bars indicate variability, representing ±1 standard deviation (SD) of model performance across bootstrap iterations, providing a measure of robustness.

Cell-segmented multiplexed immunofluorescence (mIF) data enables single-cell resolution analyses, allowing each cell to be assigned a phenotype and be counted per slide (**Figure 2A**). A defined spatial circle of influence that reflects potential contact-based and paracrine interactions can then be used to quantify each cell’s level of exposure to other cell types within its local neighborhood (**Figure 2B**). This framework enables the systematic quantification of pairwise spatial relationships between cell types, forming the basis for downstream spatial statistical and machine learning analysis.

To assess the added value of this spatial context, we first asked whether neighborhood colocalization metrics offer insights beyond those provided by traditional cell count data – that is, the number of cells of a given phenotype per slide (hereafter referred to as “counts”). To this end, we trained an OPLS model to classify regions of interest (ROIs) based on whether the percentage of PanCK^+^ cells expressing MHC class I in tumor regions was above or below 20%. This threshold was chosen as a biologically relevant round number near the middle of the distribution (median = 13%, mean = 25%) to facilitate interpretability (**Supplementary Figure S1A**). We then compared CV classification performance across models trained on counts alone, neighborhood features alone, or the combination of both (**Figure 2C**). This analysis revealed that models trained using neighborhood features alone outperformed those trained using only counts. Including both sets yielded similar, slightly improved accuracy which incorporated both counts and neighborhood features amongst VIPs. Furthermore, the variable importance in projection (VIP) scores from the combined model demonstrated that both count-based and neighborhood-derived features contributed meaningfully to classification with neighborhood scores ranking higher on the list, underscoring the added value of spatial context (**Figure 2D**).

Neighborhood colocalization data from point pattern transformations exhibit a piecewise distribution heavily skewed toward zero, reflecting the abundance of cells that lack neighbors of a given phenotype – whether common or rare (**Supplementary Figure S1B**). To assess distributional properties, focusing on nonzero values, we compared their empirical cumulative distribution function (CDF) to that of a normal distribution (**Supplementary Figure S1C**). Kolmogorov–Smirnov (KS) tests confirmed that log transformation improves the normality of the nonzero portions of the data across all phenotypes (**Supplementary Figure S1D**).

To evaluate the impact of transformation on model performance, we trained an OPLS model to classify a test cellular phenotype – i.e. IFNγ+ T cells – based on neighborhood colocalization features. We then compared models trained on raw versus log-transformed data, which revealed improved accuracy for the latter (**Supplementary Figures S2A, B**). Residual analysis – a statistical measure of prediction error – further supported this improvement (**Supplementary Figure S2C**). While the raw data produced bimodal residual distributions, log-transformed data yielded unimodal, centered residuals, indicating more consistent and unbiased model predictions. These findings support the application of log transformation to neighborhood data for downstream multivariate analyses that assume approximate normality.

These log-transformed neighborhood profiles serve as inputs for downstream multivariate modeling to uncover spatial patterns associated with cellular or tissue-level phenotypes. Although the framework is compatible with various modeling approaches, we selected OPLS methods for their balance of predictive performance and interpretability. In benchmarking experiments, OPLS-DA outperformed logistic regression and achieved comparable accuracy to random forest classifiers, which may better capture nonlinear relationships. Given the small performance difference and the advantage of interpretability, we selected OPLS-DA in the subsequent applications.

### Application 1: lymphocyte clustering predicts IFNγ expression at its focal point

**Figure 3:**
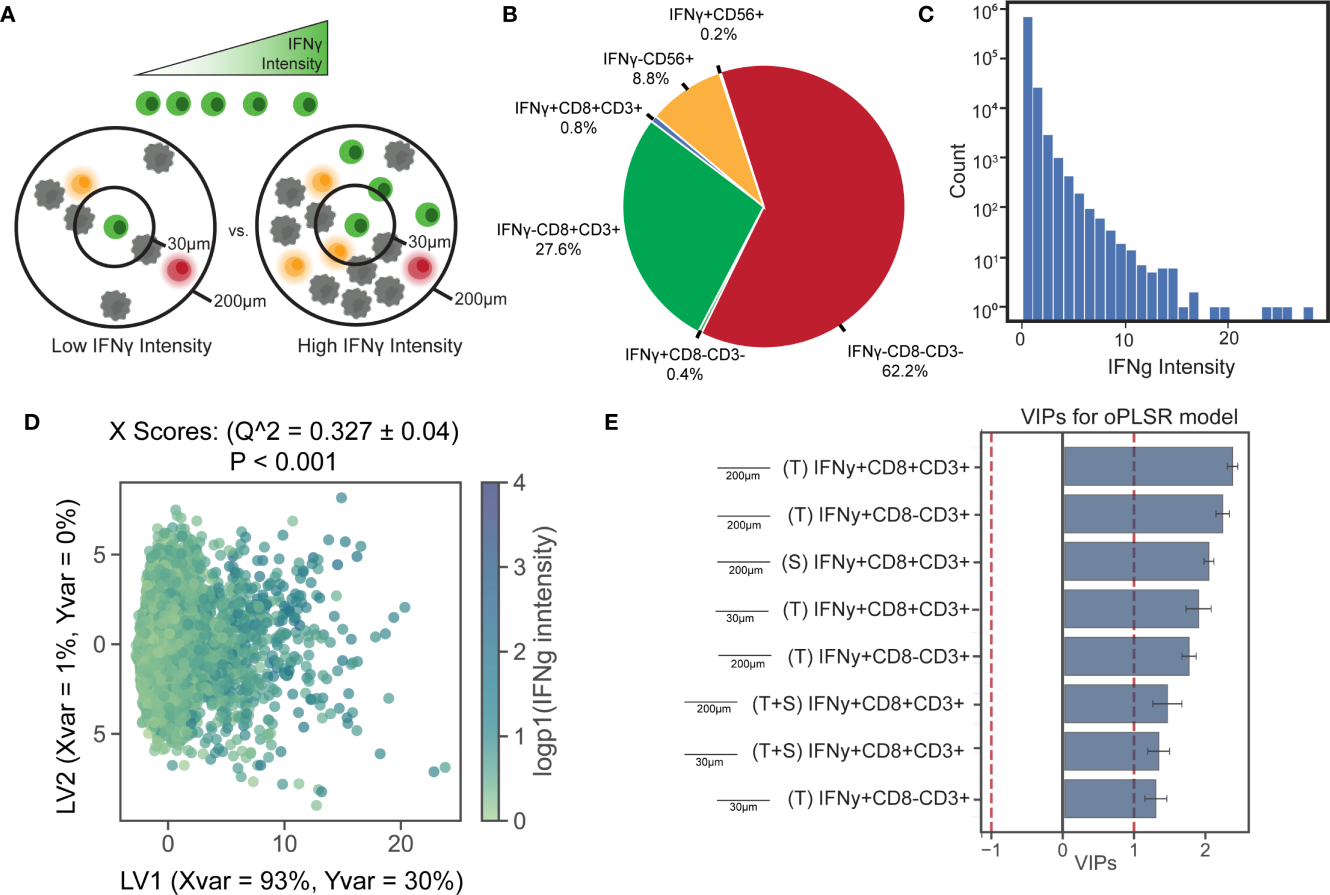
Activated T cells within paracrine-relevant distances best predict IFNγ expression in neighboring immune cells. **(A)** IFNγ intensity in immune cells (CD3^+^, CD3^-^CD56^+^) serves as a marker of lymphocyte activation. **(B)** A subset of immune cells was classified as activated based on IFNγ expression. **(C)** A histogram of IFNγ staining intensity in immune cells reveals the distribution of intensity values. Neighborhood colocalization profiles were calculated for each immune cell, and an OPLS-R model was used to predict IFNγ staining intensity based on these profiles. **(D)** Scatter plots of X scores show each immune cell as a point in the model. The model achieved a mean squared error of 0.027, a Q² of 0.31, and outperformed all 1,000 models trained on randomly permuted data. **(E)** Bar plots display VIP scores oriented by their loadings on LV1. All of the top neighborhood score features are associated with higher IFNγ intensity and are colored blue as such. Error bars show ±1 SD around mean across bootstrap iterations. Variables with VIP scores > 1 are shown, indicating above-average influence on group separation.

We applied this framework to interrogate several biological questions. In our first application, we focused on the relationship between a cell’s phenotypic or functional state and its local neighborhood profile, *i.e.* the quantity of cells of various phenotypes in its vicinity. Cytotoxicity and immune coordination first require activation of immune cells. One marker of such activation is the production of IFNγ, an inflammatory cytokine primarily produced by lymphocytes (47). The activation of T and NK cells and their subsequent IFNγ production is linked in part with their microenvironment (**Figure 3A**). IFNγ expression can be triggered by antigen recognition (48) or interactions with cognate receptors on neighboring cells, and in turn, can promote the recruitment and activation of additional immune cells. We assumed that IFNγ intensity of a cell in the images can be used as a proxy for IFNγ expression by that cell. Since we observed a broad distribution IFNγ intensity across lymphocytes (**Figures 3B, C**), we hypothesized that IFNγ intensity in T and NK cells correlates with the composition of their surrounding local neighborhood. To test this, we centered the analysis on all T cells and NK cells and quantified each individual cell’s neighbors within 30 µm and 200 µm. We then built an OPLS-Regression (OPLSR) model to predict the intensity of IFNγ staining based on these spatial features.

The top predictors of IFNγ expression included the local presence of other IFNγ^+^ lymphocytes in both tumor and stromal compartments, at distances consistent with both juxtacrine and paracrine signaling (**Figures 3D, E**, **Supplementary Figures S3A, B**). Associations within the 200 um radius – where direct cell-cell contact is less likely – had slightly stronger associations, suggesting that colocalization of activated lymphocytes may occur independently of direct contact, which is more probably within 30 um. No features were significantly negatively associated with IFNγ intensity. This pattern was visually confirmed in the images where IFNγ expressing cells formed spatial pockets (**Supplementary Figure S3C**).

These findings support a model-driven hypothesis: IFNγ expression in one lymphocyte promotes the activation and IFNγ production in other lymphocytes through a combination of direct signaling, recruitment of additional immune cells, and enhanced antigen presentation. This likely reflects a coordinated positive feedback loop that amplifies immune responses, a well-described feature of IFNγ signaling networks (49, 50), rather than direct cell-to-cell induction. Such loops are tightly regulated to prevent excessive inflammation (51).

### Application 2: IFNγ intensity in CD8^-^ CD3^+^ T cells is associated with T cell clustering

**Figure 4:**
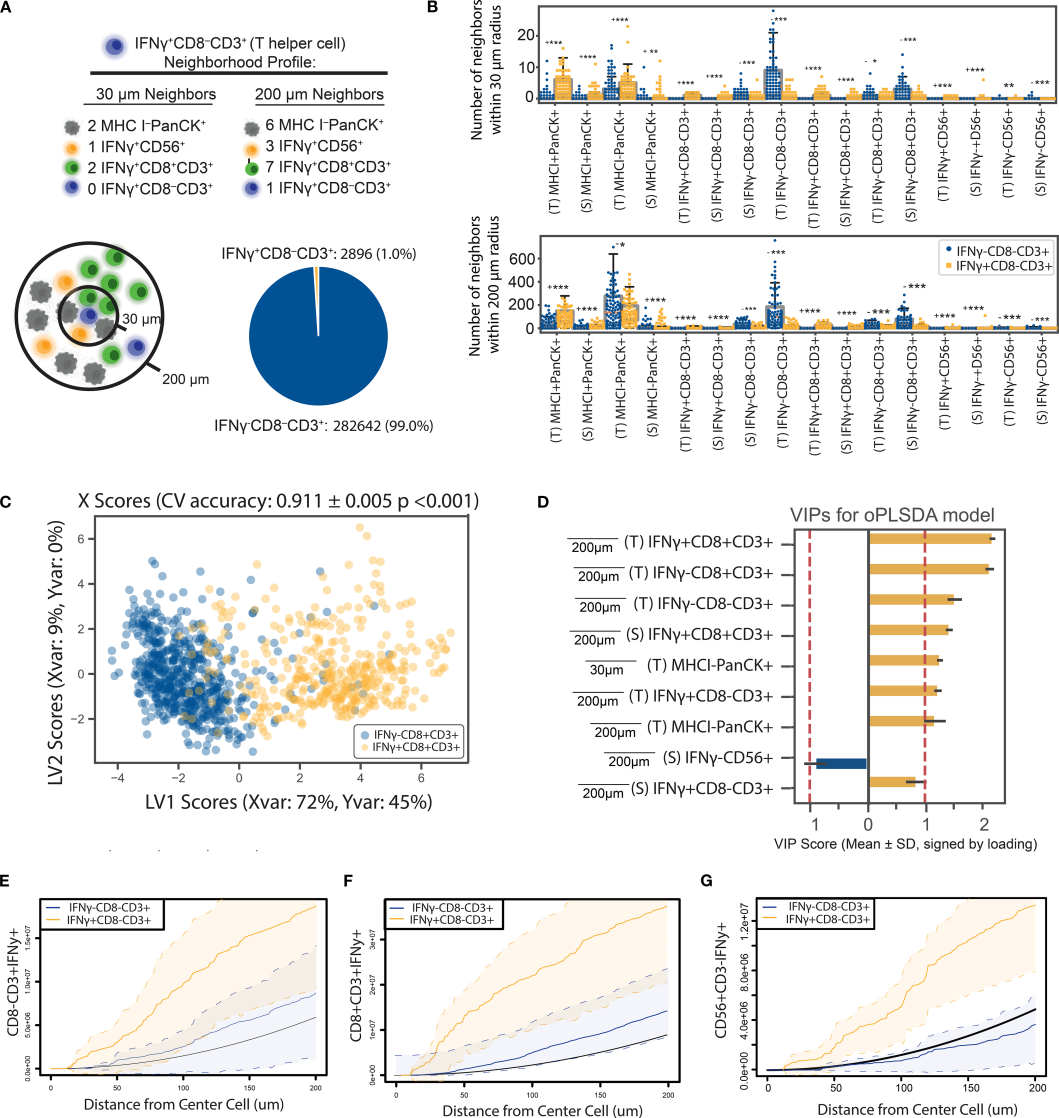
The IFNγ expression of CD8^-^ T cells are closely associated with their paracrine-range neighborhood profile. **(A)** CD8^-^ T cells were classified based on their IFNγ expression, and an OPLS-DA model was constructed using neighborhood profiles to distinguish between IFNγ^+^ and IFNγ^-^ cells. Only 1.0% of the CD8^-^ T cell population was IFN-y+ cells. The low proportion of activated CD8^-^ T cells necessitates the application of down-sampling techniques to balance the data in univariate and multivariate analysis. **(B)** Univariate comparisons revealed significant differences in neighborhood profiles of activated versus inactive CD8^-^ T cells, assessed by a two-sided Mann-Whitney test with Bonferroni correction. * p <0.05, ** p < 0.01, *** p< 1E-6, (- indicates IFNγ-CD8-CD3+ is the larger group, + indicates IFNγ+CD8-CD3+ is larger). **(C)** OPLS-DA models successfully discriminated CD8^-^ T cells based on IFNγ expression, achieving 88.0% cross-validation (CV) accuracy and outperforming 1,000 random permutations. Scatter plots show X scores, where each point represents a CD8^-^ T cell projected onto latent variables 1 and 2 (LV1 and LV2). **(D)** Bar plots of VIP scores illustrate key features associated with CD8^-^ T cells of different IFNγ expression statuses. Error bars represent ±1 standard deviation of mean across bootstrap iterations. Variables with VIP score > 1 were identified as having above-average influence on group separation. **(E)** Iterative down-sampling of center cells resulted in an average model accuracy of 0.88. **(F)** The model predicted IFNγ expression with an area under the receiver operating curve (AUROC) of 0.97, with a threshold of 0.54 used for classification. **(G)** The confusion matrix, accumulated over 5-fold CV, demonstrated a model F1 score of 0.90 and precision of 0.91. **(E–G)** Cross K-function correlation plots illustrate spatial relationships between IFNγ^+^ CD8^-^ T cells and **(E)** other IFNγ^+^ CD8^-^ T cells, **(F)** IFNγ^+^ cytotoxic T cells, and **(G)** IFNγ^+^ NK cells, across a range of radii. Dashed lines represent 95% confidence intervals, and the black line indicates the Poisson (null) distribution.

In a second application, we investigated whether the activation status of CD8^-^ CD3^+^ T cells (*i.e.*, CD4 T cells) could be predicted by their neighborhood profile. While CD8+ T lymphocytes can recognize and kill MHC class I-bearing tumor cells displaying tumor-derived antigens, CD4^+^ T cells respond with cytokine release when they detect tumor antigens displayed by MHC class II molecules on antigen-presenting cells (APCs) to coordinately regulate antitumoral immunity (52). Given their critical role in immune regulation, we hypothesized that activated (IFNγ^+^) CD4^+^ T cells exhibit distinct interaction patterns within the TME compared to IFNγ^-^ CD4^+^ T cells. Although anti-CD4 antibody was not used to identify CD4 T cells in this study, we approximated that most CD8^-^ T cells are indeed CD4^+^ T cells since T cells lacking both CD4 and CD8 comprise less than 5% of mature T cells (53). The spatial nature of these interactions was explored by examining colocalization patterns in neighborhoods extending 30-200 μm (**Figure 4A**).

To test this hypothesis, we quantified the neighborhood profiles of all CD8^-^ T cells and performed univariate comparisons between the neighborhood profiles of IFNγ^+^ and IFNγ^-^ CD8^-^ T cells (**Figure 4B**). We then constructed an OPLS-DA model with CD8^-^ T cells as center cells, IFNγ expression as the binary classifier, and neighborhood profiles as model features. The imbalance between the number of IFNγ^+^ and IFNγ^-^ CD8^-^ T cells necessitated the use of random down-sampling to address the disparity in group sizes (see Methods). Iterative down-sampling was applied to ensure all data were utilized while maintaining balanced group representation. This approach improved model precision and overall performance. Model evaluation using X scores plots demonstrated robust performance, including high accuracy and strong results in permutation testing (**Figure 4C**).

VIP scores highlighted IFNγ^+^ lymphocytes, including CD8+ T cells, CD8^-^ T cells, and NK cells (CD56^+^) at 200 um signaling distances as the strongest contributors to the separation between activated and inactive CD8^-^ T cell neighborhoods (**Figure 4D**, **Supplementary Figure S4A**). To address the imbalance in group sizes, we applied iterative random down-sampling, which ensured equal representation of IFNγ± CD8^-^ T cells in the model. The stability of this down-sampling approach was validated through 1,000 permutations, which consistently showed stable model performance (**Supplementary Figure S4B**). The model’s performance was evaluated using standard machine learning metrics, including a precision-recall curve (**Supplementary Figure S3C**), which demonstrated the model’s robustness in distinguishing between the two groups.

These results reinforce the importance of IFNγ^+^ lymphocytes in shaping the immune landscape of the TME. Further analysis of key features using Ripley’s cross K-function identified by VIP scores revealed strong spatial relationships between activated CD8^-^ T cells and other IFNγ^+^ lymphocytes, including CD8 T cells and NK cells, across a range of radii (**Figures 4E–G**). The significant and consistent separation observed in cross K-function plots supports the hypothesis that IFNγ^+^ CD8^-^ T cells have distinct neighborhood profiles that implicate their active role in the regulation and coordination of antitumoral immune responses within the TME.

Using this data and building upon existing literature, we can hypothesize mechanistic interactions at the tumor-immune interface. In NSCLC patients, IFNγ-mediated crosstalk by T cells and NK cells likely play a key role in the immune response. CD8^-^ T cells are major producers of IFNγ which activates other immune cells, including antigen presenting cells. The observed spatial relationships between CD8- T cells and other lymphocytes suggest that IFNγ secretion, especially from CD8^-^ T, cells may enhance lymphocyte clustering, thus leading to enhanced secretion of cytokines and chemokines, additional immune cell recruitment and activation which together facilitate a robust local immune response in the TME. While the current study does not directly asses IFNγ production or functional outcomes, these findings generate testable hypotheses that should be validated in future work using spatial transcriptomic analyses or functional T cell assays to assess cytokine signaling and pathway activation *in situ*.

### Application 3: tumor histologic subtype shapes MHC class I spatial relationships

**Figure 5:**
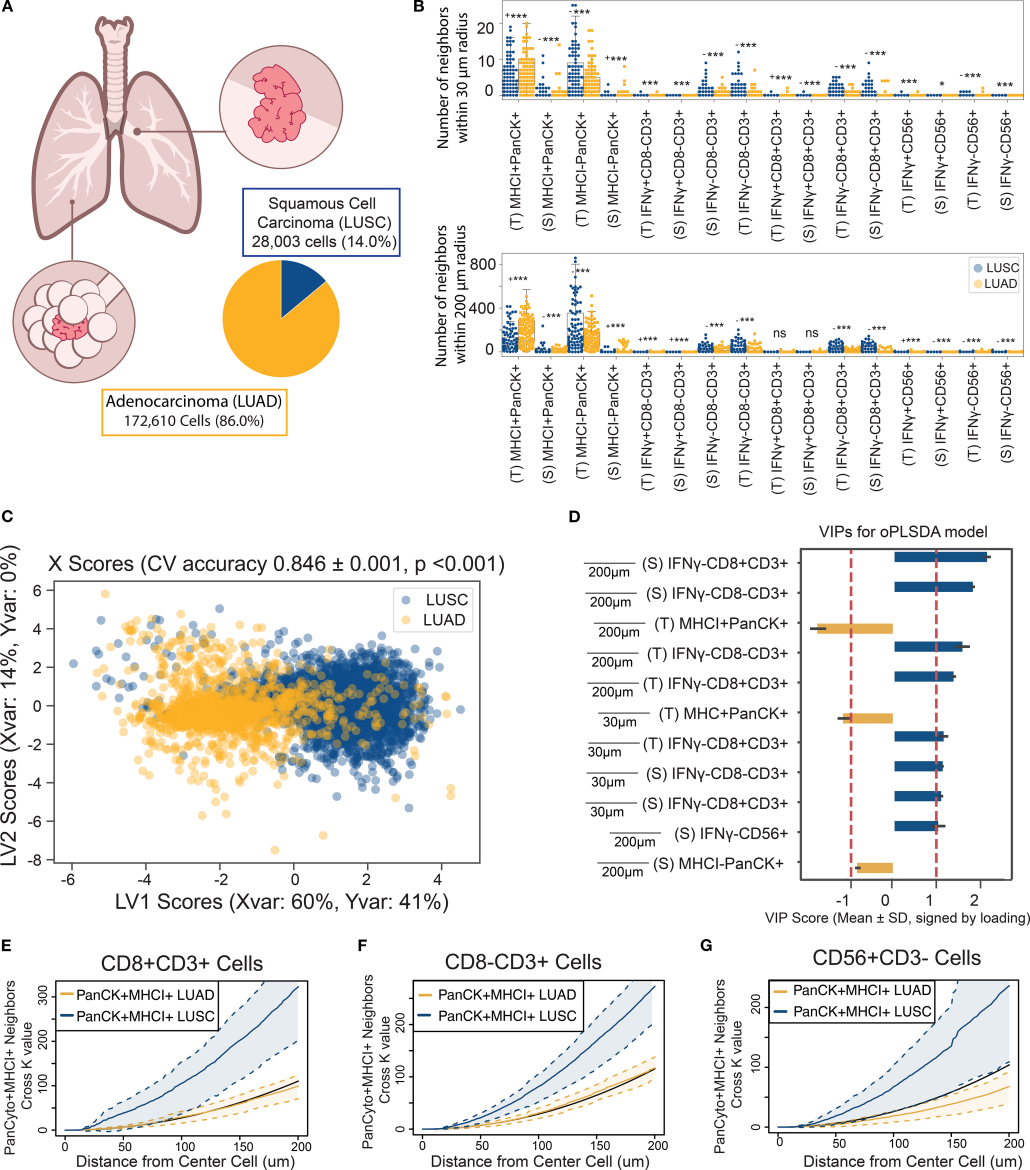
The neighborhood profile of MHC I^+^ PanCK^+^ cells distinguish their residence within LUAD or LUSC NSCLC tumors. **(A)** MHC I^+^PanCK^+^ cells were identified in LUAD and LUSC tumor regions. Group imbalance motivated the use of down-sampling techniques. **(B)** Univariate comparisons of neighborhood profiles showed significant differences between LUAD and LUSC patients, determined using a two-sided Mann-Whitney test with Bonferroni correction. * < 0.05, ** < 0.01, *** < 1E-6, (- indicates LUSC is the larger group, + indicates LUAD is larger) **(C)** An OPLS-DA model discriminated MHC I^+^ tumor cells based on their neighborhood profiles, achieving 83% cross-validation accuracy and outperforming 1,000 random permutation models (p< 0.001). Scatter plots display X scores, with each point representing a tumor cell projected onto latent variables 1 and 2 (LV1 and LV2). **(D)** Bar plots of VIP scores highlight features associated with tumor histology. Error bars represent ±1 standard deviation of mean across bootstrap iterations. Variables with |VIP score| > 1 were deemed significant contributors to class separation. **(E–G)** Cross K-function correlation plots illustrate spatial relationships between IFNγ⁺ CD8⁻ T cells and **(E)** other IFNγ⁺ CD8⁻ T cells, **(F)** IFNγ⁺ cytotoxic T cells, and **(G)** IFNγ⁺ NK cells, across a range of radii. Dashed lines represent 95% confidence intervals, and the black line indicates the Poisson (null) distribution.

Tumor histology is a critical determinant of immune landscape in NSCLC, influencing not only infiltration levels but also the spatial organization around malignant cells. Although lung adenocarcinoma (LUAD) and squamous cell carcinoma (LUSC) are both classified as NSCLC, they differ markedly in morphology, mutational burden, immunogenicity, and clinical outcomes all of which shape the tumor microenvironment in distinct ways (54, 55). Here we investigated whether these subtype specific features extend to the spatial neighborhoods of MHC class I expressing tumor cells (**Figure 5A**).

Neighborhood profiles were generated for all MHC I^+^ PanCK^+^ cancer cells residing within 30-200 µm signaling distances. Univariate analyses revealed significant differences in the composition of tumor and immune cell neighbors between LUAD and LUSC subtypes (**Figure 5B**). To quantify these differences, we developed an OPLS-DA model to predict histologic classification, LUAD or LUSC, based on the neighborhood profile of each cancer cell. The model achieved a cross-validation accuracy of 83%, outperforming 1,000 models generated with shuffled labels (**Figure 5C**). VIP scores highlighted enhanced colocalization with T lymphocytes as a distinguishing feature of LUSC-resident MHC I^+^ PanCK^+^ cells, whereas LUAD-resident cells exhibited greater colocalization with other MHC I^+^ PanCK^+^ tumor cells (**Figure 5D**, **Supplementary Figure S5A**). Due to the imbalance in sample size favoring LUAD, random down-sampling was applied to balance group sizes over 1,000 permutations, which consistently showed stable model performance (**Supplementary Figure S5B**). Precision-recall curves demonstrated consistent model performance across metrics, confirming the robustness of the analysis (**Supplementary Figure S5C**).

To further validate these findings, we used cross K-function analysis to asses spatial relationships between MHC I^+^ PanCK^+^ cells and key immune populations (IFNγ- CD8 T cells, CD8^-^ T cells, and NK cells) across a continuous range of radii (**Figures 5E–G**) which revealed significant colocalization surrounding LUSC-residing MHC I^+^ tumor cells, corroborating OPLS-DA findings and validating them across radii of interest. LUSC’s increased mutational burden and tumor-associated antigen expression likely underlie its greater immunogenicity (56). However, increased immune tolerance or suppression may limit the role of activated immune cells, contributing to poorer prognoses (55, 57). Regulatory CD4 T cells (Tregs), present in the CD8^-^CD3^+^ population, may also modulate the local immune response in LUSC tumors. Conversely, the complex morphology of LUAD may limit immune cell infiltration, but may foster more robust T cell activation, enhancing immune cell interactions within the microenvironment of MHC I^+^ cancer cells (56). However, increased immune tolerance or suppression may limit the role of activated immune cells, contributing to poorer prognoses (55, 57). Conversely, LUAD may foster more robust T lymphocyte activation, enhancing immune cell interactions within the microenvironment of MHC I^+^ tumor cells.

Together, these findings demonstrate that histologic subtype is associated with not only the molecular characteristics of tumors but also the spatial organization of immune cells within the tumor microenvironment. These patterns suggest that tumor subtype may influence or reflect immune context, motivating further investigation into the links between tumor-intrinsic features and spatial immune architecture. Such insights could eventually inform the development of more tailored therapeutic approaches, although additional and functional validation will be necessary to support subtype-specific strategies.

### Application 4: high-grade LUAD tumors are enriched for CD8^+^ T cell infiltration and MHC class I expression

Intercellular interactions in the TME are thought to influence immune regulation and thus patient outcomes. To investigate this connection, we next asked whether patterns of cellular colocalization in tumors are associated with clinical characteristics, specifically tumor grade. To this end, we compared pairwise colocalization patterns between tumors classified as high-grade and low-grade. A cancer’s grade reflects how abnormal malignant cells appear compared to healthy cells under microscopic examination. Low-grade cancer cells resemble non-malignant normal cells, are less aggressive, and are generally associated with better prognoses. In contrast, high-grade cancer cells exhibit greater deviations in appearance and organization, correlating with increased aggressiveness and worse outcomes.

**Figure 6:**
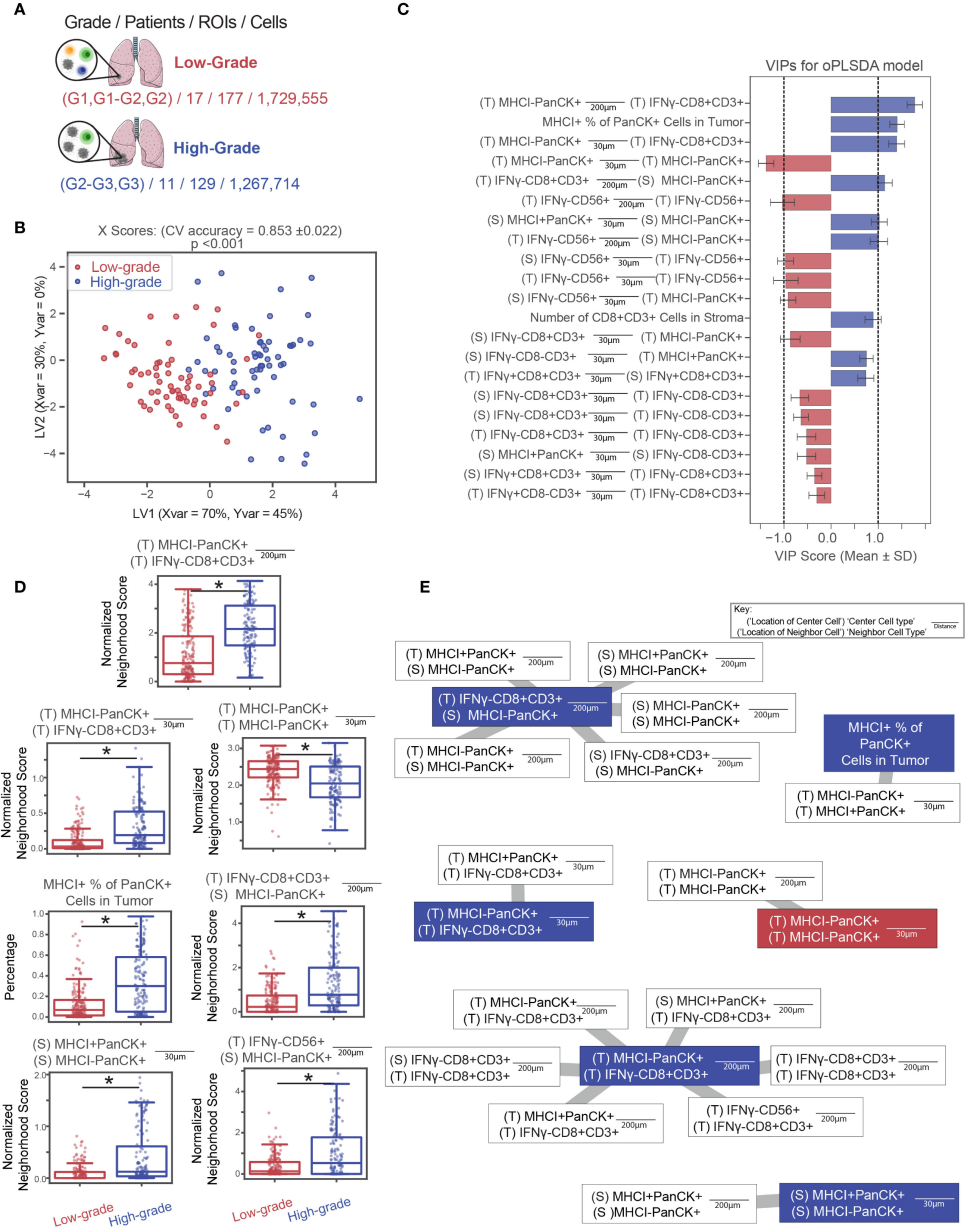
High-grade LUAD tumors are enriched for CD8^+^ T cell infiltration and MHC I expression. **(A)** ROIs were classified as low-grade or high-grade based on pathologist-defined cancer grades. **(B, C)** An OPLS-DA model was constructed to distinguish between ROIs from high- or low-grade tumors. The model achieved 76% classification accuracy in 5-fold cross-validation and outperformed all 1,000 randomly permuted models (p< 0.001). **(B)** Scatter plots of X scores depict each tumor ROI projected onto latent variables. **(C)** Bar plots of VIP scores highlight features with VIP scores > 1, indicating above-average influence on group separation. Features are artificially oriented by loadings on LV1 and colored based on association with high- or low-grade regions. Grey shading indicates stromal regions, and black shading indicates tumor regions, as defined by a random forest classifier. **(D)** Boxplots of features with |VIP score| > 1 show significant differences between groups (Mann-Whitney U test, * = p< 0.05). Error bars represent ±1 standard deviation of mean across bootstrap iterations. **(E)** Correlation networks depict non-LASSO-selected neighborhood features that were significantly correlated (Spearman r > 0.75, p< 0.05) with LASSO-selected features. Connections indicate correlation strength, and circle fill colors denote group enrichment. White outlines indicate LASSO-removed features. LV, latent variable; VIP, variable importance in projection.

To test whether higher-grade cancers exhibit distinct immune cell colocalization patterns compared to lower-grade cancers (**Figure 6A**), patient’s tumor samples were divided into two groups based on the clinical grade of their cancer as described in (35). Only LUAD patients were used to control for the differences between tumors of different histological status. Patient images were analyzed based on regions of interest (ROIs), defined as the 3mm^2^ images output by the HALO software. ROIs were labeled based on whether they originated from high- or low-grade tumors. The low-grade group contains patients labeled as G1, G1-G2, and G2 based on the American Joint Committee on Cancer (AJCC) seventh edition and was composed of 17 patients represented by 177 ROIs and 1,729,555 individual cells while the high-grade group consisted of 11 patients labeled as G2-G3, G3, and G4 represented by 129 ROIs and 1,267,714 cells. Because group sizes were comparable, no down-sampling was required.

Samples from patients in each group were characterized according to the colocalization patterns of the various cell types on an ROI-by-ROI basis. This is in contrast with the cell-by-cell basis analysis pipeline of the previous applications. Here, single cell-resolved neighborhood counts are averaged across the center cells of the same type within an ROI to result in ROI-averaged neighborhood counts as input to the downstream supervised analysis. For each ROI, cells were classified based on cell type specific stains and whether they were in the tumor or stroma, as determined by the Halo classifier. For each cell, neighborhoods were characterized according to the method described. Then for each ROI, pairwise relationships were summarized for each cell type/location as well as ROI level summaries (e.g. tumor CD8 IFNγ^+^ %, number of CD8^+^T cells in tumor regions, etc), which resulted in 528 features. To reduce model complexity and prevent overfitting, we applied LASSO regression (40) to select the most important spatial features. An OPLS-DA model was then built on these LASSO selected features to classify whether a given ROI belonged to a high- or low-grade tumor (**Figures 6B, C**, **Supplementary Figure S6**).

We assessed model stability by performing permutation testing, comparing the accuracy of the true-label model to a distribution of 1,000 models trained on randomly shuffled labels to yield an empirical p-value. This model outperformed all randomly permuted models (p<0.001) and achieved a cross-validation accuracy of 0.76. This model identified seven LASSO selected features with above-average discriminatory power VIP scores > 1 between high- and low-grade tumors, marking them as significant contributors to classification performance. Because LASSO removes features that are linearly dependent on other predictors, it may exclude features that are highly correlated with those deemed significant by downstream analysis. To ensure these correlated but excluded features are not overlooked, we used correlation networks to identify LASSO-removed features that showed strong correlations (r > 0.75) with significant features (**Figures 6D, E**).

These results indicate that high-grade tumors are characterized by CD8^+^ T cell infiltration and increased heterogeneity of tumor cell MHC class I expression. Correlation analysis between significant VIP features and non-selected features from the LASSO model revealed that, although CD8^+^ T cells were the dominant distinguishing feature, other immune populations also correlated with CD8^+^ T cell presence. We interpret these findings to suggest that high-grade tumors, characterized by greater abnormal differentiation and immunogenicity, are more likely to elicit robust T cell responses, resulting in increased infiltration by CD8^+^ T cells.

## Discussion

In this study, we present a framework for analyzing and interpreting spatially resolved single-cell data of the TME. This approach integrates an algorithm for neighborhood characterization with a robust multivariate machine learning pipeline to identify spatial relationships between cell types that are predictive of cellular state, tissue architecture, or patient outcomes. Although demonstrated here using mIF data, the framework is broadly applicable to other single-cell resolved spatial platforms, offering a flexible and interpretable approach for studying tissue organization in health and disease.

While our framework links spatial patterns to biological outcomes, findings vary in certainty. For example, the conclusion of Application 1 aligns with well-established paracrine cytokine signaling (e.g., IFNγ-mediated immune activation), as supported by recent literature. Notably, the observed distance-dependent association provides in situ evidence of paracrine cytokine activity. Although most prior studies of cytokine propagation have largely relied on in vitro or computational systems, our spatial analysis of histology data offers complementary insights. Recent experimental and modeling work has further elucidated the spatiotemporal dynamics of IL-2 and type I/II interferons – highlighting distance thresholds for effective signaling and the influence of tissue architecture – thus providing a mechanistic foundation for our findings (58–63). In contrast, the conclusions from Applications 2 and 3 are more exploratory, suggesting potential molecular characteristics and subtype-specific therapeutic strategies that warrant further experimental or multi-modal validation.

We showcase the utility of the framework in four applications: first by identifying spatial features associated with immune activation, inferred by IFNg expression (Application 1) or positivity (Application 2); distinguishing cell-type colocalization patterns across histological subtypes (Application 3); and uncovering immune spatial features predictive of tumor grade (Application 4). In each case, the pipeline accurately distinguishes between groups and identifies the most informative cell-cell colocalizations driving these distinctions. A central strength of this approach lies in its ability to operate across spatial scales, from single cells to cell neighborhoods to whole tissue regions, enabling diverse biological questions to be addressed within a unified analytical structure.

By leveraging spatial features as inputs to supervised OPLS-DA models, we uncover mechanistic insights into how cellular interactions in the TME relate to cell state and tumor microenvironment, which could be tested experimentally. This systems-level strategy supports hypothesis generation, particularly in contexts where mechanistic evidence is sparse. As spatial proteomics and transcriptomics platforms continue to evolve, this framework may serve as a foundation for exploring pathway-level drivers of immune activation or therapeutic response. To support broader adoption, we also provide methodological recommendations and implementation guidelines.

Despite its strengths, the approach has several limitations. First, spatial imaging captures a single time point and cannot resolve dynamic cellular processes, limiting interpretations to correlative rather than causative relationships. As such, mechanistic conclusions should be validated through perturbation experiments. Furthermore, imaging was performed on sliced tissue and consequently our methods focused on two-dimensional (2D) neighborhoods of cells. While this simplification does not capture the full three-dimensional (3D) architecture of the tumor microenvironment, previous studies have demonstrated that 2D spatial metrics can provide biologically meaningful insights into cellular organization when consistently applied across all samples (7–9). Future advancement in 3D image analysis can provide a more comprehensive representation of the local neighborhoods of cells and their interaction patterns. Image-based segmentation also introduces classification errors, especially in dense or multilayered tissues, where neighboring cells may be merged or misidentified. For example, closely apposed cells may be misidentified as a single cell expressing markers of multiple lineages, and cells located at different tissue depths may be difficult to resolve accurately, especially in specimens that are not uniformly one cell thick. These issues can propagate through the modeling process, making rigorous quality control and expert annotation essential.

From a computational standpoint, multivariate modeling is sensitive to input variability and may be influenced by systematic biases introduced by mislabeling, imperfect segmentation, or batch effects. Our use of feature selection and orthogonalization helps mitigate overfitting and enhances interpretability, but users should remain aware of potential pitfalls. Additionally, cytokine expression markers such as IFNγ are valuable for identifying activated immune populations but do not reveal the source of soluble signals. Therefore, spatial inference based on such markers should be interpreted with caution and, where possible, validated with orthogonal methods.

On the implementation side, this approach has additional limitations and caveats. For one, we tested the framework with a single dataset acquired with the VECTRA platform, which is limited to seven colors per panel, thus restricting the comprehensive profiling of the TME. While we focused on supervised approaches such OPLS-DA and -R as our primary modeling strategy, unsupervised and nonlinear approaches are equally valid alternatives, and reveal complimentary insights. We explored unsupervised approaches such as Uniform Manifold Approximation and Projection for Dimension Reduction (UMAP) but found them computationally intensive and poorly suited for this context, number of observations far exceeds the number of measured variables – resulting in reduced discriminatory power of the visualization. Finally, while this framework is well suited for hypothesis generation, results should be validated either in an independent cohort or experimentally.

While this framework is powerful and effective, future iterations may incorporate advances in imaging or modeling to further enhance performance. In addition, creative analytical strategies, such as modeling conditional relationships between cell types, could expand the types of questions that can be addressed beyond the scope of this study. A key strength of this pipeline is its flexibility and scalability, which make it well suited for both clinical and basic science applications. It can handle large numbers of cells and can be used to ask a wide variety of questions. The predictions of this framework offer a way to uncover important intercellular relationships across diverse disease contexts. More widespread spatial profiling and analysis with approaches like this could inform the clinical workflow, if consistent spatial relationships such as immune infiltration or spatial colocalization can be linked with response to immunotherapy. Its ability to integrate spatial data in a transparent and biologically informed manner makes it a valuable tool for both discovery and translation.

In conclusion, our study demonstrates the effectiveness of this framework in identifying crucial spatial relationships within the tumor microenvironment using a supervised machine learning approach. While the method provides unique interpretability and insight, its adaptability across spatial scales and biological systems makes it well suited for addressing a wide range of research questions. This approach holds strong potential for advancing both clinical and basic investigations into the complex organization and function of the tumor microenvironment.

## Data Availability

Publicly available datasets were analyzed in this study. This data can be found here: https://doi.org/10.18130/V3/VQFO1J.
