# Supplementary material for: A flexible systems analysis pipeline for elucidating spatial relationships in the tumor microenvironment linked with cellular phenotypes and patient-level features
Source: Front Immunol. 2025 Sep 29;16:1642527. doi: 10.3389/fimmu.2025.1642527 (PMC12515857; doi:10.3389/fimmu.2025.1642527)
Supplement: Supplementary file 1 [file Supplementaryfile1.docx]

**Supplementary Material**

**
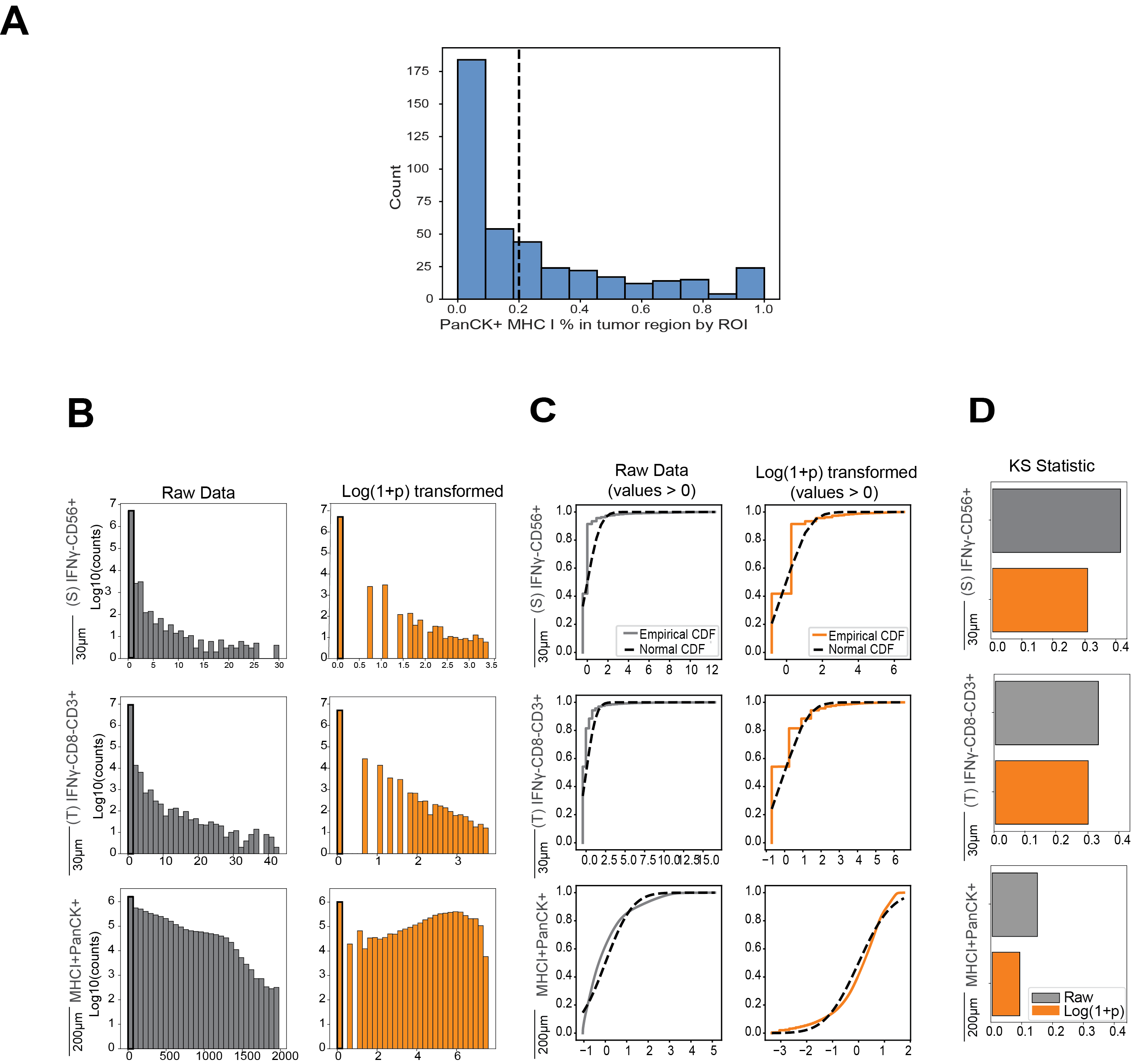
**

**Figure S1. Improving Data Normality Through Log Transformation of Features**
**(A)** Histogram showing the distribution of MHC I⁺ PanCK⁺ cells as a percentage of all PanCK⁺ cells within tumor regions, calculated on an ROI-by-ROI basis. A dotted line at 20% indicates the threshold used to classify ROIs as MHC I-high or -low. **(B)** The distribution of this transformed data is a piecewise distribution representing a zero-signal region and a signal-amplified region. **(C)** This signal-amplified region behaves in a fashion that benefits from a log transformation which improves the normality of the distribution, thereby improving it as a feature for downstream predictive models. **(D)** KS statistics for the signal-amplified region of the raw data and the log transformed data.

**
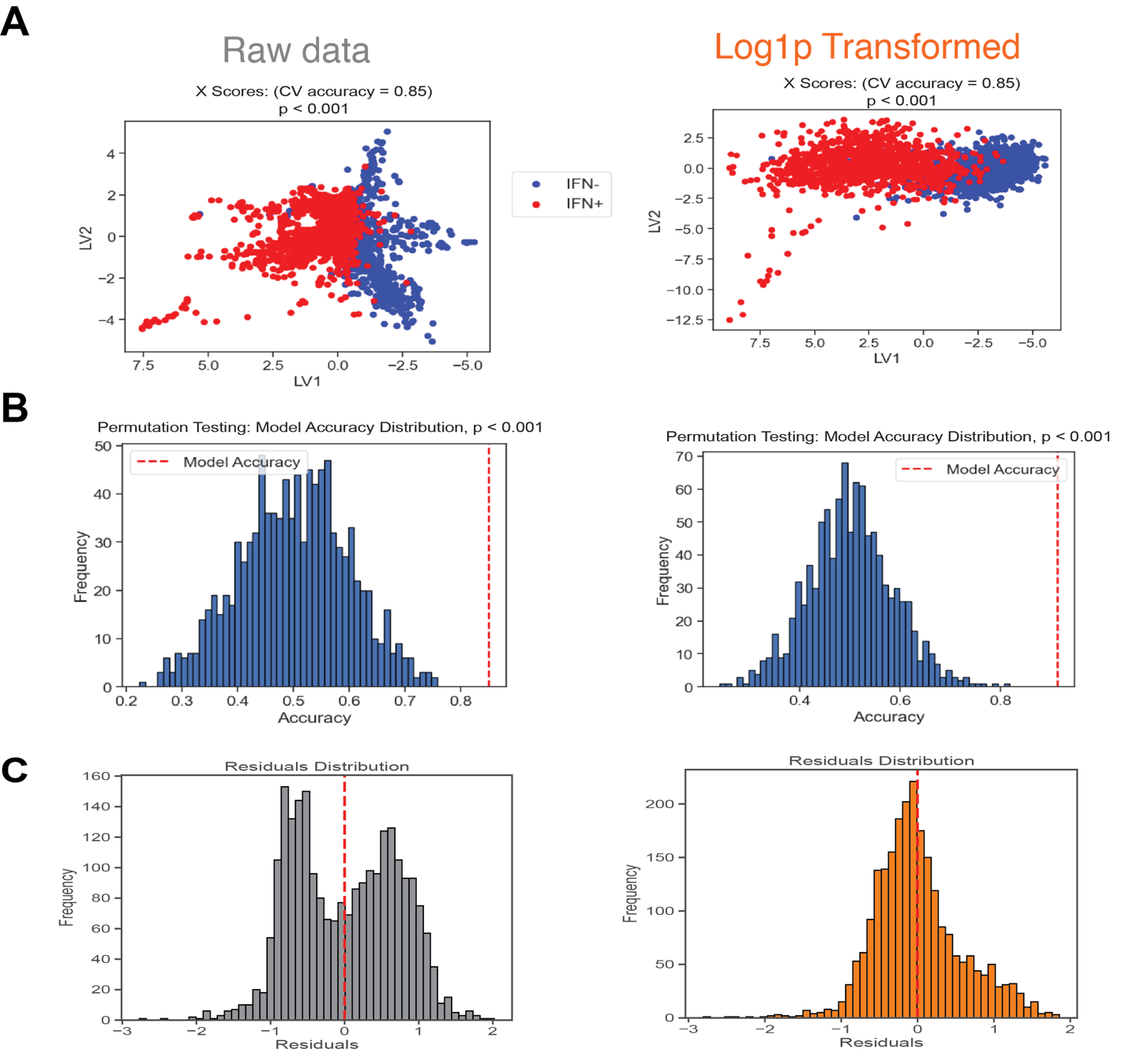
 Figure S2. Log transformation improves model performance. (A)** A total of 1,250 IFNγ⁺ and 1,250 IFNγ⁻ T cells were randomly sampled from all patients. Orthogonal partial least squares discriminant analysis (OPLSDA) models were trained on either raw or log-transformed local neighborhood profiles to predict IFNγ status. Log transformation improved classification accuracy, as shown by scores plots and **(B)** permutation test results, and **(C)** model residuals.

**
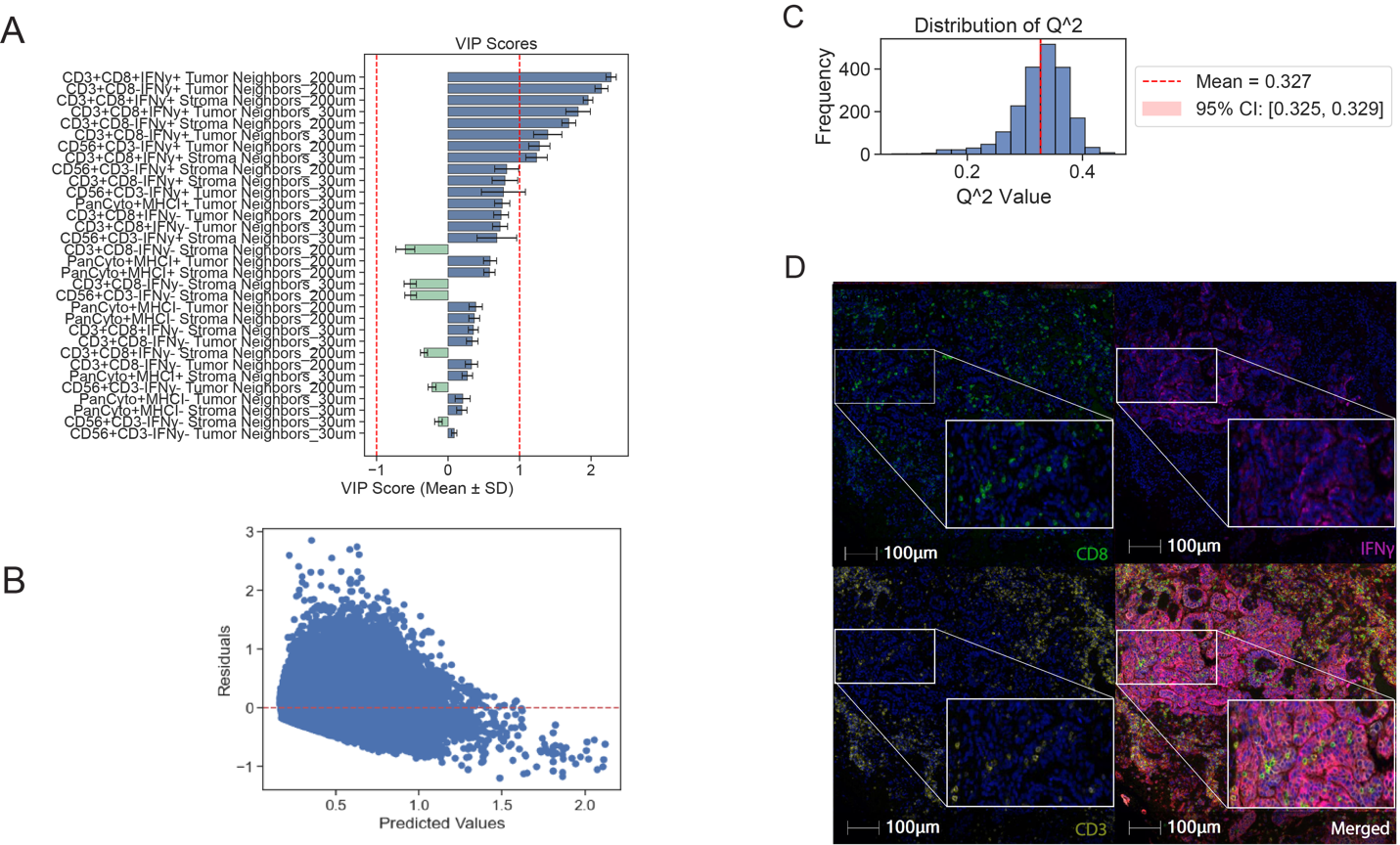
**

**Figure S3: Tumor infiltrating T cells drive IFNγ expression in lymphocytes.** This figure provides additional model metrics for the OPLSDA model of Fig. 3. **(A)** VIP scores for the full set of variables. **(B)** Residual plot shows that model error relates to the model’s tendency to overpredict the number of small values and underpredict the number of large values. **(C)** Histogram shows the distribution of Q^2^ values across bootstrap iterations, with the mean and 95% confidence intervals show. **(D)** Representative image of IFNγ and lymphocyte clustering. The boxed portion includes lymphocytes that express IFNγ as a pocket, whereas the lower left-hand portion of the image has lymphocytes that do not express IFNγ, highlighting the observation that IFNγ expression occurs in unison.

**Figure S4: The IFNγ expression of CD8⁻ T cells are closely associated with their paracrine-range neighborhood profile.** This figure provides additional model metrics for the OPLSDA model of Fig. 4. (A) VIP scores for the full set of variables. (B) Histogram shows the distribution of cross validation accuracy values across bootstrap iterations, with the mean and 95% confidence intervals show. (C) Precision-Recall curve for the model

**
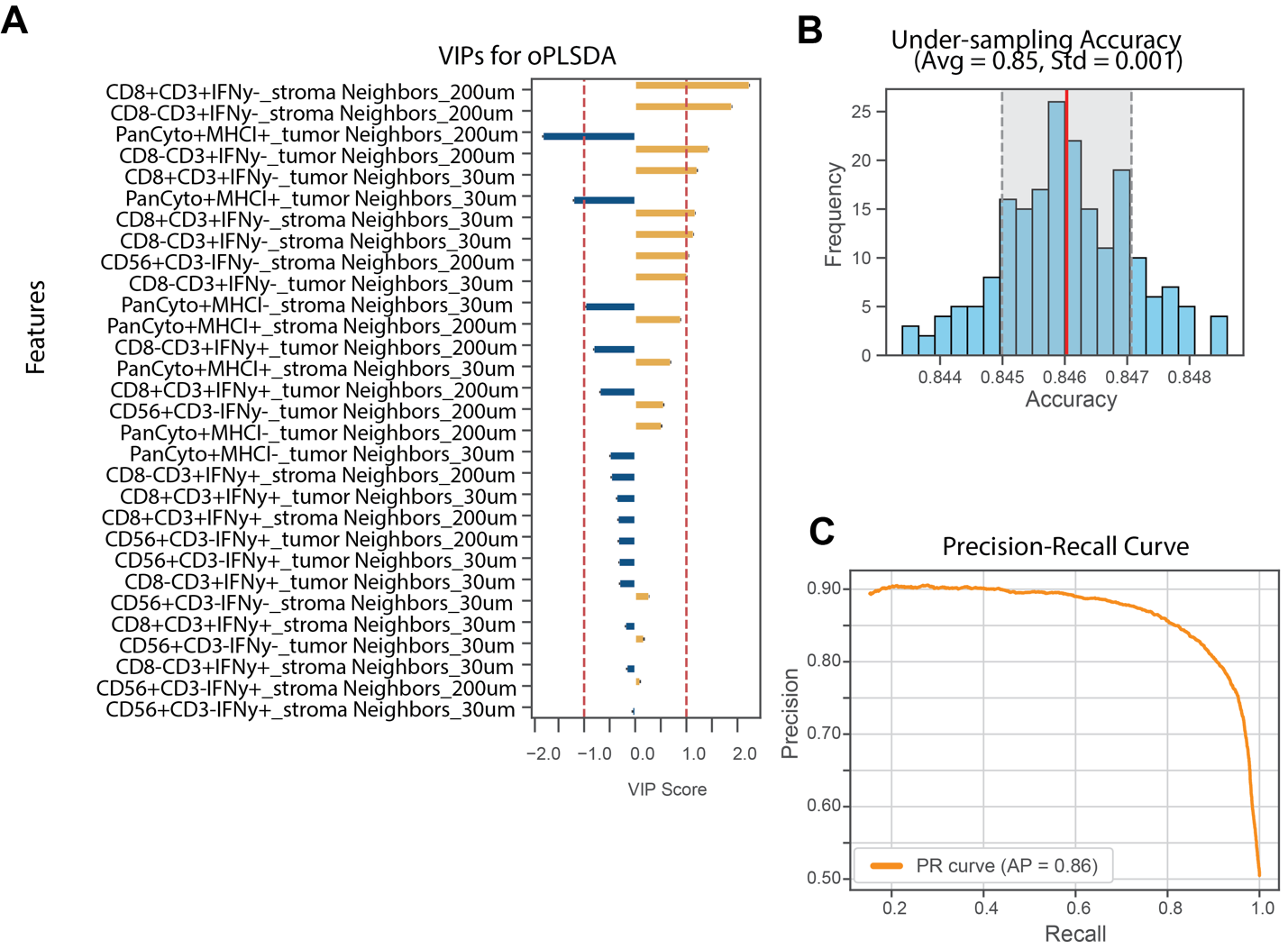
**

**Figure S5: The neighborhood profile of MHCI⁺PanCK⁺ cells distinguish their residence within LUAD or LUSC NSCLC tumors.** This figure provides additional model metrics for the OPLSDA model of Fig. 5. (A) VIP scores for the full set of variables. (B) Histogram shows the distribution of cross validation accuracy values across bootstrap iterations, with the mean and 95% confidence intervals show. (C) Precision-Recall curve for the model


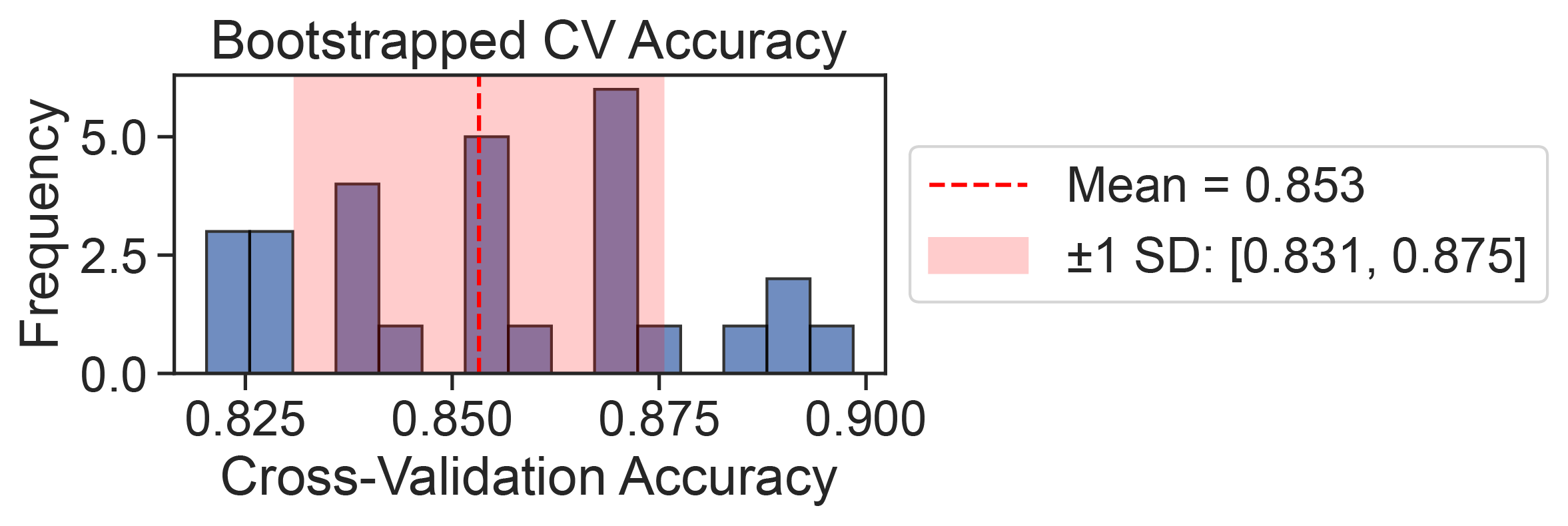


**Figure S6: High-grade LUAD tumors are enriched for CD8⁺ T cell infiltration and MHC I expression.** This figure provides additional model metrics for the OPLSDA model of Fig. 6. (A) Histogram shows the distribution of cross validation accuracy values across bootstrap iterations, with the mean and 95% confidence intervals show.
